# Electromyographic Differences in Hyoid and Superficial Muscle Activity During Dynamic Neck Movement in Individuals with Chronic Neck Pain

**DOI:** 10.3390/life16040616

**Published:** 2026-04-07

**Authors:** Hirofumi Sageshima, Ruba Albatayneh, Chipo Malambo, Dagmar Pavlů

**Affiliations:** 1 Faculty of Physical Education and Sport, Charles University, 162 52 Prague, Czech Republic; sageshima.hirofumi@ftvs.cuni.cz (H.S.); malambo@ftvs.cuni.cz (C.M.); 2 Physiotherapy Department, Faculty of Allied Medical Sciences, Applied Science Private University, Amman 11931, Jordan; ruahal@alumni.uv.es; 3 Department of Physical and Sports Education, University of Valencia, 46010 Valencia, Spain

**Keywords:** chronic neck pain, surface electromyography (EMG), muscle activity

## Abstract

Chronic neck pain (CNP) is associated with pain-related neuromuscular adaptations; however, in contrast to other superficial neck muscles, the influences of pain on hyoid muscles remain to be investigated. This study investigated how hyoid and superficial neck muscle activity differ between individuals with and without CNP during dynamic neck flexion and extension. In this observational cross-sectional, case–control study, 20 individuals with CNP and 20 sex- and age-matched asymptomatic controls were recruited. All participants performed dynamic neck flexion and extension in a crook-lying position at a controlled tempo. Surface electromyography was used to examine bilateral sternocleidomastoid (SCM), anterior scalene, upper trapezius, suprahyoid, and infrahyoid muscle activity. Normalised EMG values and their absolute phase-to-phase changes were analysed using linear mixed-effects models. A significant group × muscle interaction was observed (F = 3.34, *p* < 0.001, η^2^ = 0.04), with higher normalised EMG values in the bilateral anterior scalene (left: GMR = 1.42, *p* = 0.01; right: GMR = 1.37, *p* = 0.03) and suprahyoid muscles (left: GMR = 1.42, *p* = 0.01; right: GMR = 1.37, *p* = 0.03) in individuals with CNP. In contrast, the phase-to-phase changes did not differ between the groups. These findings suggest that individuals with CNP exhibit selective alterations in muscle activation patterns.

## 1. Introduction

Neck pain is one of the most prevalent musculoskeletal disorders worldwide and represents a leading cause of disability. The global burden has increased significantly over recent decades [1] with more than 200 million individuals affected by neck pain globally in 2020, and this number is expected to rise further in the coming decades [2].

Individuals with chronic neck pain (CNP) commonly exhibit functional impairments. This includes reduced neck muscle strength [3], altered motor control [4], altered muscle coordination [5], increased muscle coactivation [6], and alteration in the spatial distribution of muscle activity [7]. Such findings are generally interpreted as pain-related neuromuscular adaptations rather than consequences of structural weakness. According to contemporary pain adaptation theories, pain induces redistribution of muscle activity within and between muscles as a protective strategy to remove or reduce the threat to the tissues [8]. Importantly, these pain-related motor responses are highly individual and task dependent [9]. In the neck muscles, neuromuscular adaptations to pain are often characterised by altered muscle activity patterns, with reduced activation of the deep muscles and increased activation of superficial muscles, despite inconsistent findings [10,11]. Given the anterior anatomical position and potential mechanical contribution to cervical flexion, the hyoid muscles may participate in such compensatory activation patterns [11]. However, their role in the pain-related motor adaptation framework has received limited attention.

Electromyography (EMG) is a sensitive tool for investigating muscle behaviours and detecting compensatory strategies that may not be apparent during clinical examination [12,13]. In individuals with CNP, EMG has contributed substantially to the understanding of pain-related adaptations in muscle recruitment and neck motor control [14,15]. However, the majority of EMG studies have focused primarily on major cervical muscles, such as the SCM, anterior scalene and upper trapezius [16,17], and deep neck muscles, such as longus colli and rectus capitis posterior major [11,18], with comparatively little attention given to other anterior neck muscles that may also contribute to cervical movement and stability. However, limited consideration has been given to hyoid-related muscles, despite their anterior anatomical position and potential contribution to cervical motor control.

The hyoid muscles, consisting of the suprahyoid and infrahyoid muscle groups, have been studied in relation to swallowing functions [19,20]. However, due to their anterior anatomical location and attachments between the mandible and sternum via the hyoid bone, these muscles may be mechanically positioned to contribute to cervical flexion and anterior neck stabilisation. Although evidence regarding their contribution to cervical flexion moments during neck movements remains inconclusive [21], biomechanical modelling studies suggested that activation of the hyoid can generate flexion moments at the cervical spine, specifically during tasks involving head or neck flexion [22]. Despite this biomechanical potential, the functional role of the hyoid muscles during dynamic neck movements remains poorly understood, specifically in individuals with neck pain. A recent study found that hyoid muscle activity varies with body position and task demands [23]. Moreover, clinical observations suggest that individuals with chronic non-specific neck pain may increase activity of the anterior musculature, including the hyoid muscles, as a compensatory strategy when deep cervical flexor function is impaired [11]. These muscles have also been described as prone to increased tone or spasm in the presence of cervical dysfunction [24]. Experimental studies further demonstrated that hyoid muscle activation is highly variable among individuals [25], indicating that further investigation is required. Despite growing evidence of altered cervical muscle activity in CNP, only a limited number of studies have explicitly examined hyoid muscle EMG activity. Previous studies have predominantly examined the hyoid muscles in relation to swallowing or biomechanical modelling, rather than during active cervical movement tasks in individuals with CNP.

Therefore, the primary aim of the present study was to investigate the characteristics of hyoid muscle activity during dynamic neck flexion and extension in individuals with CNP compared with asymptomatic controls. As a secondary aim, the activity of selected superficial neck muscles (SCM, anterior scalene, and upper trapezius) was examined to characterise muscle-specific recruitment patterns during the task. We hypothesised that individuals with CNP would demonstrate altered activity patterns of the hyoid muscles during dynamic neck flexion and extension, potentially accompanied by increased recruitment of superficial neck flexors, reflecting modified motor control strategies.

## 2. Methods

### 2.1. Study Design and Setting

This observational cross-sectional case–control study was reviewed and approved by the Ethics Committee at the Faculty of Physical Education and Sport, Charles University, Czechia (EK 284/2021), an institutional review body authorised to assess research involving human participants, including health-related and medical research. The study involved standardised functional movement tasks and maximal voluntary contractions performed under controlled laboratory conditions without therapeutic intervention or alteration of ongoing treatment. According to institutional regulations, the study was classified as non-interventional observational research. All procedures were conducted in accordance with the Declaration of Helsinki. Data were collected from January 2022 to December 2023 at a laboratory within the Faculty of Physical Education and Sport, Charles University, Czechia. Each participant attended a single testing session in the laboratory. All participants provided written informed consent before the study. The reporting of EMG acquisition, processing, and analysis procedures adhered to the Consensus for Experimental Design in Electromyography (CEDE) guidelines [26]. The CEDE-Check, developed to promote consistency, transparency, and reproducibility in the reporting of surface EMG methodology outcomes, served as the reporting framework.

### 2.2. Participants

A total of 40 participants were recruited from the Faculty of Physical Education and Sport, Charles University, Czechia, through social media announcements and distributed information leaflets. The sample comprised 20 individuals with non-specific CNP and 20 asymptomatic controls, matched individually to CNP participants by sex and age (±5 years). Sample size estimation was informed by prior electromyography studies comparing individuals with chronic neck pain and asymptomatic controls, which reported moderate-to-large between-group differences in neck muscle activity during comparable laboratory protocols [17]. A prior sample size estimation was performed using G*Power 3.1 for Windows for a repeated-measures ANOVA (within–between interaction) design, with group as the between-subject factor and phase as the within-subject factor, assuming a medium effect size (f = 0.25) was selected based on findings from a previous study between-group differences in normalised EMG amplitude in chronic neck pain populations [17]. This previous study reported a Cohen’s d of approximately 0.62, corresponding to an effect size of f ≈ 0.31 in a two-group design [17]. A conservative medium effect size (f = 0.25), an α of 0.05, a β power of 0.8, two groups (CNP and asymptomatic controls), and two phases (neck flexion and extension), which required 34 participants. Finally, we recruited 40 participants, assuming a data loss due to poor signal quality or participant withdrawal. As G*Power software does not provide analytical solutions for a priori power estimation in linear mixed models (LMM), the sample size in this study was estimated using a repeated-measures ANOVA framework. This approach is commonly used to obtain a pragmatic and conservative estimate of the sample size required to detect fixed effects in repeated-measures designs [27].

The inclusion criteria for the CNP group were as follows: (i) self-reported non-specific neck pain, defined as pain localised around the neck region without radiation beyond the shoulder, persisting for more than three months; (ii) scored ≥5 out of 50 in the neck disability index (NDI) questionnaire (described in the later section) [28] and at least 10 out of 100 in the visual analogue scale (VAS) (also described in the later section) [29], indicating at least mild disability; and (iii) absence of clinical signs or diagnoses indicating specific pathology, including cervical radiculopathy, myelopathy, fracture, infection, or inflammatory arthropathy.

The inclusion criteria for the control group were as follows: (i) no current neck or shoulder pain; (ii) no self-reported history of neck or shoulder pain requiring clinical management or activity modification within the preceding 12 months; and (iii) current neck pain intensity rated ≤10/100 on a visual analogue scale.

The exclusion criteria for both groups were as follows: (i) presence of systemic medical conditions, including rheumatic or neuromuscular disorders; (ii) history of spinal deformity or spinal surgery; (iii) cervical radiculopathy or myelopathy; (iv) history of whiplash or traumatic neck injury within the preceding 12 months; (v) pregnancy; (vi) receipt of any form of healthcare treatment for neck pain within the six months preceding participation; and (vii) current use of muscle relaxants or medications known to affect neuromuscular function. All eligibility criteria were assessed via telephone or email screening before laboratory attendance, with verbal confirmation obtained on the day of testing.

### 2.3. Questionnaires

Pain intensity was rated using a 100 mm VAS, where 0 represented “no soreness at all” and 100 meant “extreme soreness” [30]. The reliability of the VAS in chronic musculoskeletal pain has been established [31]. Additionally, all participants completed the NDI to quantify disability related to neck symptoms [28]. The NDI comprises 10 items, which are measured on a 6-point scale from 0 (no disability) to 50 (full disability). The numeric responses for each item were summed to yield a score ranging from 0 to 50. The reliability of the NDI has been established, and it is well-correlated with the VAS and the numeric rating scale (NRS) for pain [32]. The questionnaires were administered using paper forms before electrode placement.

### 2.4. Electromyography

EMG signals were recorded using a 16-channel telemetry system (TELEmyo DTS EMG sensors, Noraxon, AZ, USA) with the following specifications: baseline noise < 1 µV RMS; input impedance > 100 MΩ; common mode rejection ratio (CMRR) > 100 dB; gain = 500; bandwidth 10–500 Hz; sampling rate 1500 Hz; 16-bit resolution. Disposable, self-adhesive silver/silver chloride (Ag/AgCl) bipolar surface electrodes were used in the experiment. The figure-of-eight shaped adhesive measured 40 mm × 22 mm, with circular electrode contacts 10 mm in diameter and an inter-electrode distance of 20 mm centre-to-centre. The raw EMG signals were stored using the biomechanical analysis software MR 3.8.30 (Noraxon U.S.A. Inc., Scottsdale, AZ, USA) and then analysed using custom scripts (Mathworks Matlab 2023a).

**Figure 1:**
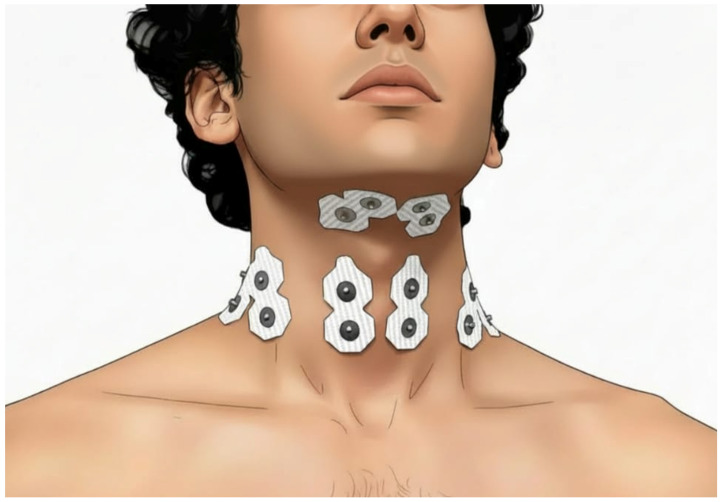
Locations of surface electrodes over the participant’s bilateral sternocleidomastoid (SCM), anterior scalene, suprahyoid, and infrahyoid muscles. Electrodes placed over the upper trapezius are not visible, as the figure shows an anterior view.

EMG was recorded bilaterally from the SCM, anterior scalene, upper trapezius, suprahyoid, and infrahyoid muscles. Before attaching the electrodes, the skin was prepared by shaving hair, if necessary, and then cleaning the skin with an alcohol swab. Electrode placements followed SENIAM (surface EMG for a non-invasive assessment of muscles) recommendations and previous studies [33,34,35,36]: (i) SCM electrodes were placed at one-third of the distance from the sternal notch toward the mastoid process; (ii) anterior scalene electrodes were positioned posterior to the lateral border of the clavicular head of the SCM, at the level of the cricoid cartilage, with placement verified via palpation during deep inspiration; (iii) upper trapezius electrodes were placed at 50% of the distance along the line from the acromion to the spinous process of the C7 vertebra; (iv) suprahyoid electrodes were positioned midway between the inferior border of the mandible and the thyroid cartilage; and (v) infrahyoid electrodes were placed midway between the superior attachment on the body of the hyoid bone and the inferior attachment on the manubrium and clavicle. The locations of surface electrodes are shown in Figure 1. A reference electrode was placed over the right clavicle.

Cross-talk from adjacent muscles is common in the neck region, where small muscles are attached [37]. To minimise cross-talk, electrode locations were carefully selected by activating each muscle in turn. For example, cross-talk from the SCM was evaluated by rotating the head to the contralateral side before attaching electrodes to the infrahyoid muscles. Additionally, to minimise variability, electrode placement for all participants was performed by the same trained investigator following a standardised protocol.

### 2.5. Task and Study Procedures

Participants performed two standardised maximal voluntary isometric contractions (MVCs) for EMG amplitude normalisation: neck flexion in the crook-lying position and shoulder shrug while sitting in a chair, each against manual resistance applied by an investigator. Each contraction was held for 5 s with 2 min of rest between trials, and standardised verbal encouragement (“Push, you can do it”) was provided to enhance performance [38]. To reduce variability, MVC measurements for all participants were conducted by the same investigator. The reporting of the MVC measurement adhered to the CEDE project: Amplitude normalisation matrix [39].

Following the MVC assessment, participants assumed a crook-lying position (supine with hips and knees flexed, feet flat on the plinth, and arms resting at their sides). The starting position was standardised with the head resting comfortably on the plinth in a neutral position, maintaining the natural cervical lordosis. Participants were instructed to maintain relaxed breathing throughout the task and to keep their gaze fixed on a point on the ceiling.

Participants performed five repetitions of controlled neck flexion and extension movements. Each repetition consisted of: (i) a flexion phase, lifting the head from the starting position until the chin approached the chest (participant’s comfortable maximum range of motion); and (ii) an extension phase, lowering the head back to the starting position. Movement tempo was controlled using a digital metronome set at 60 beats per minute (one beat per second), with each phase lasting 4 s (4 beats). Participants were cued with “up-2-3-4, down-2-3-4” to maintain tempo. A 30 s rest period was provided between repetitions. Prior to data collection, participants completed one practice trial to familiarise themselves with the movement pattern and tempo. Movement quality and tempo adherence were visually monitored by the investigator throughout testing, with verbal cueing provided when participants deviated from the prescribed tempo.

### 2.6. Data Analysis

All data analyses were performed using custom scripts (Mathworks Matlab 2023a). The second to fourth movement repetitions were used for analysis to reduce variability associated with task familiarisation and end-task fatigue. Prior to analysis, electrocardiogram (ECG) artefacts were attenuated using an adaptive subtraction technique [40], as biological artefacts are common in EMG recordings of the neck and upper trunk [37]. A notch filter was also applied to eliminate 50 Hz power line interference.

EMG signals from each channel were then processed as follows: (i) removal of DC offset; (ii) band-pass filter (20–450 Hz, fourth-order Butterworth); (iii) full-wave rectification; (iv) computing RMS using a 200 ms window. Additionally, to permit comparison of relative muscle activation between groups, RMS values were normalised to the peak RMS value calculated over a 200 ms window during the MVC trials. Normalisation to MVC is widely used in musculoskeletal and pain studies to facilitate between-subject and between-group comparisons [15,41]. The CEDE states that MVC normalisation may be biased in populations with pain if participants are unable to produce an actual maximal effort [39]. Although we acknowledge this potential limitation, MVC was used for EMG normalisation due to its established reliability [42]. In this study, most participants with CNP reported mild symptoms, and none were unable to complete MVC testing because of pain. MVC procedures were standardised across groups, and identical instructions and testing conditions were applied to minimise systematic bias.

### 2.7. Statistical Analysis

Statistical analyses were performed using R version 4.3.1 (R Core Team, Vienna, Austria, 2023) and RStudio version 2023.06.0+421 (Posit Software, Boston, MA, USA). Demographic characteristics between the CNP and control groups were compared using independent-samples *t*-tests when assumptions of normality and homogeneity of variance, assessed using the Shapiro–Wilk and Levene’s tests, respectively, were met; otherwise, Mann–Whitney U tests were applied. Raw MVC RMS amplitudes were also compared between groups to assess potential effects of pain-related inhibition on EMG normalisation. Data are presented as mean ± SD, and effect sizes are reported as Hedges’ g.

Analyses were conducted using normalised EMG values from both the right and left sides of each muscle. In addition to the bilateral analysis, a side-matched comparison was performed, comparing the most painful side in the CNP group with the right and dominant sides of the control group, consistent with previous pain-related EMG studies [43,44]. The right side was selected as a consistent anatomical reference to avoid variability associated with random side selection. Furthermore, the experimental task consisted of controlled neck flexion–extension, a symmetrical movement in the sagittal plane that is not expected to load one side preferentially. To verify that the use of the right side as a reference would not introduce systematic bias, an additional analysis comparing EMG amplitudes between the left and right sides within the control group was performed. An analysis comparing the most painful side in the CNP group with the dominant side in the control group was also conducted to examine potential dominance-related effects. Linear mixed-effects model (LMM) analyses were performed using the R package lme4 [45]. This approach was chosen because it is well-suited to repeated-measures experimental designs and allows simultaneous modelling of fixed experimental effects (group, muscle, and phase) while accounting for within-participant correlations via random effects [46]. Before analysis, normalised RMS values were log-transformed as inspection of residual distributions indicated right-skewness and heteroscedasticity. The following model was used:

normalised RMS~group × phase × muscle + (1|subject)

Group (CNP and control) was used as a between-subject factor, whereas phase (flexion and extension) and muscle (SCM, anterior scalene, trapezius, supra- and infrahyoid) were used as within-subject factors. Random intercepts for subject were included to account for inter-individual differences in baseline muscle activity and the non-independence of repeated measurements within participants. Models including random slopes for phase (1 + phase|subject) were tested but excluded due to convergence issues and lack of improvement in model fit. Therefore, the final analyses retained a random-intercept structure.

To examine between-group differences in the magnitude of phase-related changes independent of direction, absolute phase differences in normalised RMS values were calculated as the absolute difference between flexion and extension phases for each muscle. These absolute difference values were log-transformed to improve normality and homoscedasticity, and then were also analysed using a separate LMM with group and muscle as fixed effects and a random intercept for subject:

absolute phase difference~group × muscle + (1|subject)

Random slopes were evaluated but not retained for this model for the same reasons described above.

Model assumptions were assessed through: (i) visual inspection of residual plots (fitted vs. residuals, Q-Q plots); (ii) Shapiro–Wilk tests on model residuals; and (iii) homoscedasticity checks using residual plots stratified by group and phase. Assumptions were considered adequately met if no systematic patterns were evident and residuals approximated normality.

Post hoc comparisons were conducted using estimated marginal means (emmeans package) [47] with Holm–Bonferroni correction. Group differences are presented as geometric mean ratios (GMR) after back-transformation. The GMR represents the multiplicative effect size for between-group comparisons. A GMR > 1 indicates higher normalised EMG values in the CNP group compared with the controls, whereas a GMR < 1 represents lower normalised EMG values in the CNP. Percentage differences were derived from these ratios to facilitate the interpretation of multiplicative between-group effects. Standardised effect sizes were also reported as Hedges’ g [48].

Exploratory associations between symptom severity (VAS, NDI) and normalised EMG amplitudes were assessed using Spearman correlations within the CNP group. To minimise multiple testing, analyses were restricted to muscles showing significant between-group differences. Statistical significance was set at α = 0.05.

## 3. Results

### 3.1. Participants

All 40 participants (20 CNP and 20 asymptomatic controls) completed the study. Their demographic characteristics are detailed in Table 1. No significant differences in height and weight were observed. The individuals with CNP presented with a higher level of disability, showing 10.25 ± 3.74 in NDI, which was characterised as a mild disability [28]. Additionally, the individuals in the CNP group reported a current pain intensity of 3.04 ± 1.62, which was characterised as mild pain [49].

### 3.2. Electromyographic Measures

Raw MVC amplitudes were compared between the CNP and control groups to examine whether pain-related inhibition could influence EMG normalisation. No significant differences in MVC amplitudes were observed between groups for any of the examined muscles (all *p* > 0.05), suggesting that reduced maximal effort in the CNP group was unlikely to bias EMG normalisation systematically.

The LMM revealed a significant main effect of phase (F = 43.78, *p* < 0.001, η^2^ = 0.06) and a significant main effect of muscle (F = 788.45, *p* < 0.001, η^2^ = 0.91), indicating phase- and muscle-dependent differences in normalised EMG amplitude. The main effect of group was not significant (F = 2.02, *p* = 0.163, η^2^ = 0.05).

**Figure 2:**
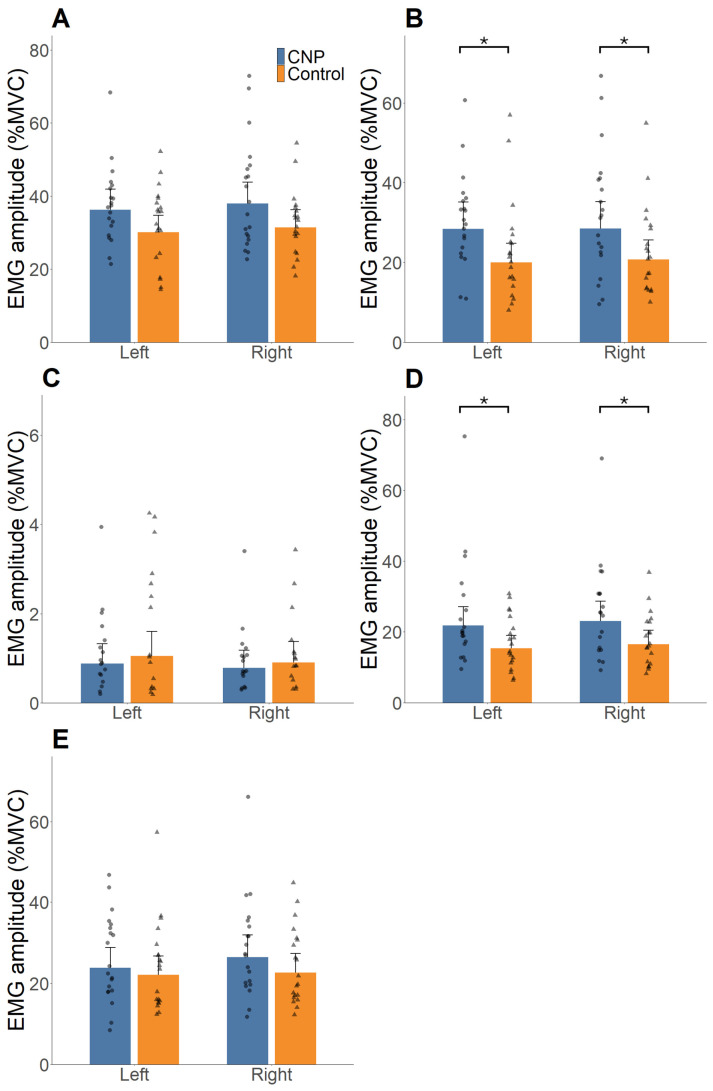
Average EMG amplitude expressed as percentage of maximal voluntary contraction (%MVC) of the left and right sternocleidomastoid (SCM) (**A**), anterior scalene (**B**), upper trapezius (**C**), suprahyoid (**D**), and infrahyoid (**E**) muscles. Bars represent back-transformed model-estimated marginal means, and points represent individual participant means averaged across Phase 1 and 2. Blue bars indicate the chronic neck pain (CNP) group, and orange bars indicate the asymptomatic control group. Error bars indicate the upper bound of the 95% confidence interval. Post hoc pairwise comparisons were performed using Holm–Bonferroni correction; * *p* < 0.05.

A significant group × muscle interaction was observed (F = 3.34, *p* < 0.001, η^2^ = 0.04). Post hoc analyses with Holm–Bonferroni adjustment demonstrated higher normalised EMG amplitude in the CNP group compared with controls for the bilateral anterior scalene (left: GMR = 1.42, 95% CI [1.07–1.87], *p* = 0.01, Hedge’s g = 0.87; right: GMR = 1.37, 95% CI [1.04–1.82], *p* = 0.03, Hedge’s g = 0.79), corresponding to approximately 42% and 37% higher normalised EMG values on the left and right sides of the muscles, respectively. Similarly, higher normalised EMG amplitude in the bilateral suprahyoid macules was observed in the CNP group (left: GMR = 1.36, 95% CI [1.03–1.80], *p* = 0.03, Hedge’s g = 0.77; right: GMR = 1.34, 95% CI [1.01–1.77], *p* = 0.04, Hedge’s g = 0.73), corresponding to approximately 36% and 34% greater normalised EMG values on the left and right sides, respectively, compared with controls. No significant group × phase or phase × muscle interactions were identified (F = 1.09, *p* = 0.163, η^2^ < 0.01; F = 1.43, *p* = 0.170, η^2^ = 0.02, respectively), and no group × phase × muscle interaction was observed (F = 0.09, *p* = 0.997, η^2^ < 0.01). The percentage differences reported above were derived from geometric mean ratios estimated from the log-transformed mixed-effects model and therefore may not correspond directly to visual differences between the plotted marginal means shown in Figure 2.

Similar results were observed in the comparison between the most painful side in the CNP and the right side in the control group. A significant group × muscle interaction was observed (F = 3.76, *p* = 0.005, η^2^ = 0.04), along with the main effects of phase and muscle (F = 23.67, *p* < 0.001, η^2^ = 0.07; F = 1126.13, *p* < 0.001, η^2^ = 0.93, respectively). Post hoc analyses with Holm–Bonferroni adjustment revealed that a significant difference in normalised EMG values in the scalene muscle for the most painful side in the CNP group compared with the controls (GMR = 1.33, 95% CI [1.03; 1.71], *p* = 0.03, Hedge’s g = 0.77), indicating 33% higher normalised EMG amplitude in the scalene for individuals with CNP. Similarly, a significant difference in the suprahyoid muscle between groups (GMR = 1.34, 95% CI [1.04; 1.74], *p* = 0.02, Hedge’s g = 0.81), indicating 34% higher normalised EMG amplitude in the suprahyoid for the CNP participants compared with the controls. In addition to comparison between the most painful side in the CNP group and the dominant side in the controls, a significant group × muscle interaction was observed (F = 4.09, *p* = 0.003, η^2^ = 0.05), along with the main effects of phase and muscle (F = 20.83, *p* < 0.001, η^2^ = 0.06; F = 1062.62, *p* < 0.001, η^2^ = 0.93, respectively). Post hoc analyses with Holm-Bonferroni adjustment revealed that a significant difference in normalised EMG values in the scalene muscle for the most painful side in the CNP group compared with the controls (GMR = 1.31, 95% CI [1.01; 1.70], *p* = 0.04, Hedge’s g = 0.75), indicating 33% higher normalised EMG amplitude in the scalene for individuals with CNP. Similarly, a significant difference in the suprahyoid muscle between groups (GMR = 1.33, 95% CI [1.03; 1.73], *p* = 0.02, Hedge’s g = 0.79), indicating 33% higher normalised EMG amplitude in the suprahyoid for the CNP participants compared with the controls. To confirm that selecting the right side as a reference did not introduce systematic bias, EMG amplitudes between the left and right sides within the control group were also compared. No significant differences were observed for any muscle (all *p* > 0.05), confirming left–right equivalence in controls.

To evaluate whether symptom severity influenced muscle activation, exploratory Spearman correlation analyses were conducted within the CNP group, which revealed no significant associations between pain intensity (VAS) and normalised EMG amplitudes of the bilateral suprahyoid or anterior scalene muscles (ρ = −0.32 to 0.04, all *p* > 0.05). Similarly, no significant associations were observed between NDI scores and normalised EMG amplitudes (ρ = −0.13 to 0.09, all *p* > 0.05).

Analysis of absolute phase-to-phase changes revealed a significant main effect of muscle (F = 56.15, *p* < 0.001, η^2^ = 0.61). No significant main effect was observed between the CNP and control groups (F = 2.94, *p* = 0.09, η^2^ = 0.07). The group × muscle interaction was not significant (F = 0.48, *p* = 0.89, η^2^ = 0.01), indicating absolute phase-related changes across muscles did not differ between groups. When comparing the most painful side in the CNP group with the right side in the control group, a significant main effect of muscle on absolute phase-to-phase changes was observed (F = 106.89, *p* < 0.001, η^2^ = 0.77). However, no significant main effect and group × muscle interaction were detected in this comparison (F = 0.74, *p* = 0.39, η^2^ = 0.02; F = 0.31, *p* = 0.87, η^2^ < 0.01, respectively). Similarly, when comparing the most painful side in the CNP group with the dominant side in the control group, a significant effect of muscle was observed (F = 97.67, *p* < 0.001, η^2^ = 0.76). In contrast, no significant main effect and group × muscle interaction were detected (F = 0.85, *p* = 0.36, η^2^ = 0.05; F = 0.42, *p* = 0.79, η^2^ = 0.01, respectively).

## 4. Discussion

In this observational cross-sectional, case–control study, we aimed to determine how hyoid and superficial neck muscle activity differs during dynamic neck flexion and extension in individuals with CNP compared with asymptomatic controls. The findings of this study revealed that individuals with CNP demonstrated specific muscle alterations in normalised EMG amplitude, with a significant group × muscle interaction. This indicates higher normalised EMG amplitude in the bilateral anterior scalene and suprahyoid muscles during a dynamic neck flexion–extension movement. However, no differences between the groups were observed for the SCM, upper trapezius, and infrahyoid muscles across the tasks. Similarly, higher normalised EMG amplitude for the scalene and suprahyoid muscles was observed when comparing the most painful side in the CNP group with the right side or dominant side in the control group. Furthermore, the examination of absolute phase-to-phase variations indicated that the temporal adaptation of muscle activity was maintained, with no significant differences across groups or group × muscle interaction.

The observation of increased normalised EMG amplitude in the anterior scalene in individuals with CNP is consistent with previous findings reporting increased activity in the superficial neck flexor muscles during motor-control tasks, such as the cranio-cervical flexion test [50]. Increased activity of superficial neck muscles in individuals with CNP is interpreted as a compensatory strategy for altered neck motor control. Superficial neck muscles are considered to contribute substantially to force generation [51]. Previous studies have reported that individuals with CNP neck exhibit higher superficial muscle stiffness in asymptomatic individuals [52], which is associated with reduced movement amplitude and velocity, likely helping minimise the effects of perturbations [8]. However, muscle stiffness was not assessed in this study, so that no direct inferences can be made regarding its relationship with the observed differences in normalised EMG amplitude. These findings should be interpreted in terms of relative muscle activity rather than mechanical muscle properties. Furthermore, increased superficial muscle activity may reflect impaired deep neck flexor function, indicating the altered motor control of the cervical spine due to a long-lasting pain condition [4,11]. In addition to pain-related motor adaptation, increased relative muscle activation in the anterior scalene may reflect an enhanced stabilisation strategy during dynamic head movement, providing local stability to the cervical spine [53]. Furthermore, the scalene muscles are secondary respiratory muscles, and individuals with neck pain exhibit increased scalene muscle activity along with the SCM, which is associated with respiratory dysfunction [54]. These factors were considered to increase normalised EMG amplitude in the anterior scalene. However, these mechanisms cannot be distinguished within this experimental study design and warrant further investigation. In contrast to the current findings, a previous study reported lower anterior scalene activity during neck flexion and extension in individuals with CNP compared to the controls, which was interpreted as pain-related inhibition [17]. However, the previous study employed a different task involving neck flexion and extension movements in the standing position, suggesting that superficial muscle activity patterns may be task-dependent.

Despite increased relative muscle activation in the anterior scalene, no differences in the SCM and upper trapezius between the groups were observed. The SCM has the largest flexion moment arm and is a primary force generator during neck flexion [21,51]; it was highly contracted during neck flexion and extension in individuals with and without CNP. In contrast, although the upper trapezius has a neck extension moment arm [21] and has been frequently examined in previous studies, it is considered a stabilising muscle rather than a prime mover during neck extension [55]. In this study, the normalised EMG amplitude in the upper trapezius remained relatively low across neck movements, which may explain the absence of differences between groups for this muscle.

Additionally, higher relative muscle activation in the suprahyoid muscles was observed in individuals with CNP. Although these muscles are associated with orofacial function rather than cervical movement, their activity, along with that of infrahyoid muscles, has been consistently reported during neck flexion [23,36] and during isometric neck contractions [56]. However, no studies have compared the hyoid muscle activity between individuals with and without CNP. Therefore, the involvement of the suprahyoid muscles may reflect adaptive changes in motor control associated with pain, characterised by a redistribution of muscle activity to protect the affected body segment and potentially enhancing cervical spine stability [57]. Importantly, pain-related motor adaptations are unlikely to be uniform across muscles or individuals [25,58], arising from selective modifications of motor unit activity across muscle regions rather than a global increase in activation in the presence of pain. This interpretation supports the presence of selective alterations in muscle recruitment strategies rather than a generalised increase in neck muscle activity. In contrast to the suprahyoid muscle, no significant differences in normalised EMG amplitude between the groups were detected for the infrahyoid muscle. A previous study reported that the sternohyoid muscle, a component of the infrahyoid muscle, acted as a synergist of neck flexor muscles and demonstrated strong coordination with the SCM during neck flexion [56]. Additionally, the sternohyoid is recruited during neck flexion-related tasks requiring maximal effort [36]. Given the relatively high normalised EMG amplitude in the SCM during both neck flexion and extension, the infrahyoid muscle also showed high relative activation across the task. This sustained level of activation may have accounted for the lack of differences between the groups for this muscle.

Regarding analysis of absolute phase-to-phase changes, while a muscle-dependent effect was observed, indicating that some muscles exhibited higher phase-to-phase variability, no significant group differences and muscle × group interaction were observed. Participants in this experiment performed dynamic neck flexion and extension movements at a controlled tempo in a crook-lying position. In this position, the head was constantly under gravitational loading, and movement relies on controlled concentric contraction of the cervical flexors during the flexion phase and eccentric contraction during the extension phase. An external extension moment acts on the head for most of the movement phase, resulting in activity of cervical flexor muscles during both the neck flexion and extension [23,59]. This sustained contraction across the phases may have reduced phase-specific modifications of muscle activity, thereby limiting the detection of group differences in the absolute phase-to-phase changes.

### 4.1. Clinical Relevance

The selective involvement of the anterior scalene and suprahyoid muscles during a dynamic neck movement may indicate the importance of evaluating muscle coordination and movement patterns, rather than relying solely on global muscle strength or overall activation level. These findings align with clinical evaluation of movement strategies during functional tasks, such as controlled head lifting and neck flexion, when disproportionate activation of the hyoid or superficial neck muscles may be evident. However, given that pain-related motor adaptations vary considerably between individuals, some participants with CNP in this study demonstrated increased normalised EMG amplitude in the hyoid muscles, whereas others exhibited greater relative muscle activation in the SCM and anterior scalene muscles. Importantly, the present study was observational and does not imply that specific muscles are directly targeted for strength or intervention. Instead, they suggest that altered coordination within anterior neck musculature may be present in some individuals with CNP, warranting individualised clinical evaluation.

### 4.2. Methodological Considerations

In this study, several limitations must be acknowledged. First, participants with chronic neck pain were relatively young. They reported mild pain intensity and mild disability, which may limit the generalisability of the findings to individuals with more severe symptoms or long-standing symptoms. Second, we acknowledged that the CEDE indicates that MVC normalisations may introduce bias [39]. Although participants in the present study reported mild pain and completed MVC procedures, the equivalent of maximal neural drive between groups cannot be definitively confirmed. To evaluate the possibility that pain-related inhibition could influence EMG normalisation, comparisons of raw MVC amplitudes between groups were performed in each muscle and no systematic reductions in the CNP group were observed (all *p* > 0.05), suggesting that pain-related inhibition during MVC testing was unlikely to influence EMG normalisation substantially. Additionally, the findings did not exhibit a global increase in normalised EMG amplitude across all muscles in the CNP group, showing muscle-specific differences. Taken together, although MVC normalisation remains a methodological limitation, the absence of widespread positive pain-EMG associations and the lack of a global amplitude increase reduce the likelihood that the observed group differences are primarily attributable to normalisation bias.

Third, artefacts, including cross-talk from adjacent muscles, may have influenced the surface EMG signals recorded from the neck muscles. Due to the anatomical proximity and muscle redundancy of anterior neck muscles [60], complete separation of activity from adjacent muscles cannot be guaranteed when using surface electrodes [61]. Therefore, cross-talk from adjacent muscles may have influenced the recorded signals, despite adherence to established guidelines and procedures to minimise signal contamination. Consequently, the findings in this study should be interpreted with consideration of this limitation. Fourth, the experimental task imposed relatively high and sustained muscle activity demands, which may have limited the ability to detect group differences, specifically in the analysis of absolute phase-to-phase changes. To evaluate the altered motor control between individuals with and without CNP, lower-load tasks, such as the cranio-cervical flexion test, may be more sensitive. Nevertheless, differences in normalised RMS values between groups were observed in the present study, indicating altered muscle activity despite the demanding task conditions. Fifth, a cross-sectional design prevents causal interference regarding the relationship between pain and altered muscle activation patterns. Sixth, a priori power estimation was conducted using a repeated-measures ANOVA framework because analytical solutions for mixed-effects models are not readily available in standard software. While this approach provides a reasonable approximation [27], statistical power for higher-order interactions in mixed-effects models may differ from ANOVA-based estimates. Finally, kinematic measures were not included in this study. Future studies incorporating kinematic analyses and longitudinal study designs may provide further insight into alterations of motor control in individuals with CNP.

## 5. Conclusions

In the present study, individuals with CNP demonstrated higher normalised EMG amplitude in the bilateral anterior scalene and suprahyoid muscles during neck flexion and extension, with moderate-to-large effect sizes, compared with asymptomatic controls. These findings indicate selective alterations in relative muscle activation patterns during dynamic neck movements in individuals with CNP.

## Figures and Tables

**Table 1:** Demographic characteristics of participants in both CNP and control groups (mean ± SD).

Characteristics	CNP (n = 20)	Control (n = 20)	*p*	Effect Size (Hedges’ g)
Sex (female: male)	11:9	11:9	-	-
Age, year	24.4 ± 2.5	24.6 ± 2.6	0.803	0.08
Height, m	1.72 ± 0.1	1.71 ± 0.1	0.665	0.14
Weight, kg	66.2 ± 13.6	69.0 ± 10.4	0.484	0.22
BMI	22.1 ± 2.45	23.6 ± 2.79	0.088	0.54
NDI *	10.25 ± 3.74	0.9 ± 1.17	<0.001	3.31
VAS *	3.04 ± 1.62	0.00 ± 0.00	<0.001	NA
Most painful side (left: right)	6:14	NA	-	-

CNP: chronic neck pain; BMI: body mass index; NDI: neck disability index; VAS: visual analogue scale; NA: not applicable. * Significant difference between groups.

## Data Availability

The original contributions presented in this study are included in the article. Further inquiries can be directed to the corresponding author.

## References

[1] Zhu Y., Yang Y., Yi H., Jiang T. (2025). Global, regional, and national burden of disease in neck pain: Statistical analysis of incidence, prevalence, and DALYS, with projections to 2036. Eur. Spine J..

[2] Wu A.-M., Cross M., Elliott J.M., Culbreth G.T., Haile L.M., Steinmetz J.D., Hagins H., A Kopec J., Brooks P.M., Woolf A.D. (2024). Global, regional, and national burden of neck pain, 1990–2020, and projections to 2050: A systematic analysis of the Global Burden of Disease Study 2021. Lancet Rheumatol..

[3] Miranda I.F., Wagner Neto E.S., Dhein W., Brodt G.A., Loss J.F. (2019). Individuals with Chronic Neck Pain Have Lower Neck Strength Than Healthy Controls: A Systematic Review with Meta-Analysis. J. Manip. Physiol. Ther..

[4] Woodhouse A., Vasseljen O. (2008). Altered motor control patterns in whiplash and chronic neck pain. BMC Musculoskelet. Disord..

[5] Falla D., Farina D., Dahl M.K., Graven-Nielsen T. (2007). Muscle pain induces task-dependent changes in cervical agonist/antagonist activity. J. Appl. Physiol..

[6] Lindstrøm R., Schomacher J., Farina D., Rechter L., Falla D. (2011). Association between neck muscle coactivation, pain, and strength in women with neck pain. Man. Ther..

[7] Barbero M., Falla D., Mafodda L., Cescon C., Gatti R. (2016). The Location of Peak Upper Trapezius Muscle Activity During Submaximal Contractions is not Associated with the Location of Myofascial Trigger Points: New Insights Revealed by High-density Surface EMG. Clin. J. Pain.

[8] Hodges P.W. (2011). Pain and motor control: From the laboratory to rehabilitation. J. Electromyogr. Kinesiol..

[9] Hodges P.W., Smeets R.J. (2015). Interaction between pain, movement, and physical activity: Short-term benefits, long-term consequences, and targets for treatment. Clin. J. Pain.

[10] Bonilla-Barba L., Florencio L.L., Rodríguez-Jiménez J., Falla D., Fernández-de-Las-Peñas C., Ortega-Santiago R. (2020). Women with mechanical neck pain exhibit increased activation of their superficial neck extensors when performing the cranio-cervical flexion test. Musculoskelet. Sci. Pract..

[11] Jull G., Falla D. (2016). Does increased superficial neck flexor activity in the craniocervical flexion test reflect reduced deep flexor activity in people with neck pain?. Man. Ther..

[12] Dirito A.M., Abichandani D., Jadhakhan F., Falla D. (2024). The effects of exercise on neuromuscular function in people with chronic neck pain: A systematic review and meta-analysis. PLoS ONE.

[13] Veen E.J.D., Koorevaar C.T., Verdonschot K.H.M., Sluijter T.E., de Groot T., van der Hoeven J.H., Diercks R.L., Stevens M. (2021). Compensatory Movement Patterns Are Based on Abnormal Activity of the Biceps Brachii and Posterior Deltoid Muscles in Patients with Symptomatic Rotator Cuff Tears. Clin. Orthop. Relat. Res..

[14] Tsang S.M.H., Szeto G.P.Y., Xie Y.F., Lee R.Y.W. (2018). Association of electromyographic activation patterns with pain and functional disability in people with chronic neck pain. Eur. J. Appl. Physiol..

[15] Nobe R., Yajima H., Takayama M., Takakura N. (2022). Characteristics of Surface Electromyograph Activity of Cervical Extensors and Flexors in Nonspecific Neck Pain Patients: A Cross-Sectional Study. Medicina.

[16] Figas G., Kostka J., Pikala M., Kujawa J.E., Adamczewski T. (2024). Analysis of Clinical Pattern of Musculoskeletal Disorders in the Cervical and Cervico-Thoracic Regions of the Spine. J. Clin. Med..

[17] Lascurain-Aguirrebeña I., Newham D.J., Galarraga-Gallastegui B., Critchley D.J. (2018). Differences in neck surface electromyography, kinematics and pain occurrence during physiological neck movements between neck pain and asymptomatic participants. A cross-sectional study. Clin. Biomech..

[18] Röijezon U., Jull G., Djupsjöbacka M., Salomoni S.E., Hodges P.W. (2021). Deep and superficial cervical muscles respond differently to unstable motor skill tasks. Hum. Mov. Sci..

[19] Chang W.H., Chen M.H., Liu J.F., Chung W.L., Chiu L.L., Huang Y.F. (2023). Surface Electromyography for Evaluating the Effect of Aging on the Coordination of Swallowing Muscles. Dysphagia.

[20] Oh J.C. (2024). Changes in the Activation Level of the Floor of the Mouth Muscles during Pressing and Swallowing Tasks According to the Degree of Tongue Pressure. Dysphagia.

[21] Vasavada A.N., Li S., Delp S.L. (1998). Influence of muscle morphometry and moment arms on the moment-generating capacity of human neck muscles. Spine.

[22] Mortensen J.D., Vasavada A.N., Merryweather A.S. (2018). The inclusion of hyoid muscles improve moment generating capacity and dynamic simulations in musculoskeletal models of the head and neck. PLoS ONE.

[23] Sageshima H., Pavlů D., Dvořáčková D., Pánek D. (2022). Onset Timing of Hyoid Muscles Activation during Cervical Flexion Is Position-Dependent: An EMG Study. Life.

[24] Janda V. (1986). Some aspects of extracranial causes of facial pain. J. Prosthet. Dent..

[25] Gizzi L., Muceli S., Petzke F., Falla D. (2015). Experimental Muscle Pain Impairs the Synergistic Modular Control of Neck Muscles. PLoS ONE.

[26] Besomi M., Devecchi V., Falla D., McGill K., Kiernan M.C., Merletti R., van Dieën J.H., Tucker K., Clancy E.A., Søgaard K. (2024). Consensus for experimental design in electromyography (CEDE) project: Checklist for reporting and critically appraising studies using EMG (CEDE-Check). J. Electromyogr. Kinesiol..

[27] Kumle L., Võ M.L., Draschkow D. (2021). Estimating power in (generalized) linear mixed models: An open introduction and tutorial in R. Behav. Res. Methods.

[28] Vernon H., Mior S. (1991). The Neck Disability Index: A study of reliability and validity. J. Manip. Physiol. Ther..

[29] Turner J.A., Franklin G., Heagerty P.J., Wu R., Egan K., Fulton-Kehoe D., Gluck J.V., Wickizer T.M. (2004). The association between pain and disability. Pain.

[30] Bijur P.E., Silver W., Gallagher E.J. (2001). Reliability of the visual analog scale for measurement of acute pain. Acad. Emerg. Med..

[31] Boonstra A.M., Schiphorst Preuper H.R., Reneman M.F., Posthumus J.B., Stewart R.E. (2008). Reliability and validity of the visual analogue scale for disability in patients with chronic musculoskeletal pain. Int. J. Rehabil. Res..

[32] Saltychev M., Pylkäs K., Karklins A., Juhola J. (2024). Psychometric properties of neck disability index—A systematic review and meta-analysis. Disabil. Rehabil..

[33] Falla D., Dall’Alba P., Rainoldi A., Merletti R., Jull G. (2002). Location of innervation zones of sternocleidomastoid and scalene muscles—A basis for clinical and research electromyography applications. Clin. Neurophysiol..

[34] Falla D., Jull G., O’Leary S., Dall’Alba P. (2006). Further evaluation of an EMG technique for assessment of the deep cervical flexor muscles. J. Electromyogr. Kinesiol..

[35] Hermens H.J., Freriks B., Disselhorst-Klug C., Rau G. (2000). Development of recommendations for SEMG sensors and sensor placement procedures. J. Electromyogr. Kinesiol..

[36] O’Leary S., Falla D., Jull G., Vicenzino B. (2007). Muscle specificity in tests of cervical flexor muscle performance. J. Electromyogr. Kinesiol..

[37] Sommerich C.M., Joines S.M., Hermans V., Moon S.D. (2000). Use of surface electromyography to estimate neck muscle activity. J. Electromyogr. Kinesiol..

[38] McNair P.J., Depledge J., Brettkelly M., Stanley S.N. (1996). Verbal encouragement: Effects on maximum effort voluntary muscle action. Br. J. Sports Med..

[39] Besomi M., Hodges P.W., Clancy E.A., Van Dieën J., Hug F., Lowery M., Merletti R., Søgaard K., Wrigley T., Besier T. (2020). Consensus for experimental design in electromyography (CEDE) project: Amplitude normalization matrix. J. Electromyogr. Kinesiol..

[40] Abbaspour S., Fallah A. (2014). Removing ECG Artifact from the Surface EMG Signal Using Adaptive Subtraction Technique. J. Biomed. Phys. Eng..

[41] Cid M.M., Januario L.B., Zanca G.G., Mattiello S.M., Oliveira A.B. (2018). Normalization of the trapezius sEMG signal—A reliability study on women with and without neck-shoulder pain. Braz. J. Phys. Ther..

[42] Burden A. (2010). How should we normalize electromyograms obtained from healthy participants? What we have learned from over 25 years of research. J. Electromyogr. Kinesiol..

[43] Schomacher J., Boudreau S.A., Petzke F., Falla D. (2013). Localized pressure pain sensitivity is associated with lower activation of the semispinalis cervicis muscle in patients with chronic neck pain. Clin. J. Pain.

[44] Sjörs A., Larsson B., Dahlman J., Falkmer T., Gerdle B. (2009). Physiological responses to low-force work and psychosocial stress in women with chronic trapezius myalgia. BMC Musculoskelet. Disord..

[45] Bates D., Mächler M., Bolker B., Walker S. (2015). Fitting Linear Mixed-Effects Models Using lme4. J. Stat. Softw..

[46] Boccia G., Martinez-Valdes E., Negro F., Rainoldi A., Falla D. (2019). Motor unit discharge rate and the estimated synaptic input to the vasti muscles is higher in open compared with closed kinetic chain exercise. J. Appl. Physiol..

[47] Lenth R.V., Piaskowski J. (2025). Emmeans: Estimated Marginal Means, aka Least-Squares Means. https://rvlenth.github.io/emmeans/.

[48] Zieliński G. (2026). Getting to Know Pain Effect Sizes—Guidelines for Effect Size and Sample Size in Global Pain Research. Arch. Phys. Med. Rehabil..

[49] Boonstra A.M., Schiphorst Preuper H.R., Balk G.A., Stewart R.E. (2014). Cut-off points for mild, moderate, and severe pain on the visual analogue scale for pain in patients with chronic musculoskeletal pain. Pain.

[50] Müller-Thyssen-Uriarte J., Lucha-López M.O., Hidalgo-García C., Sánchez-Rodríguez R., Vicente-Pina L., Ferrández-Laliena L., Vauchelles-Barré P., Tricás-Moreno J.M. (2024). Electromyographic Activity of Cervical Muscles in Patients with Neck Pain and Changes After Dry Needling: A Narrative Review. J. Clin. Med..

[51] Cheng C.H., Chien A., Hsu W.L., Chen C.P., Cheng H.Y. (2016). Investigation of the Differential Contributions of Superficial and Deep Muscles on Cervical Spinal Loads with Changing Head Postures. PLoS ONE.

[52] Taş S., Korkusuz F., Erden Z. (2018). Neck Muscle Stiffness in Participants with and Without Chronic Neck Pain: A Shear-Wave Elastography Study. J. Manip. Physiol. Ther..

[53] Sremakaew M., Treleaven J., Jull G., Vongvaivanichakul P., Uthaikhup S. (2021). Altered neuromuscular activity and postural stability during standing balance tasks in persons with non-specific neck pain. J. Electromyogr. Kinesiol..

[54] Cefalì A., Santini D., Lopez G., Maselli F., Rossettini G., Crestani M., Lullo G., Young I., Dunning J., de Abreu R.M. (2025). Effects of Breathing Exercises on Neck Pain Management: A Systematic Review with Meta-Analysis. J. Clin. Med..

[55] Lecompte J., Maisetti O., Guillaume A., Skalli W., Portero P. (2007). Agonist and antagonist EMG activity of neck muscles during maximal isometric flexion and extension at different positions in young healthy men and women. Isokinet. Exerc. Sci..

[56] Siegmund G.P., Blouin J.S., Brault J.R., Hedenstierna S., Inglis J.T. (2007). Electromyography of superficial and deep neck muscles during isometric, voluntary, and reflex contractions. J. Biomech. Eng..

[57] Hodges P.W., Tucker K. (2011). Moving differently in pain: A new theory to explain the adaptation to pain. Pain.

[58] Hug F., Dernoncourt F., Avrillon S., Thorstensen J., Besomi M., van den Hoorn W., Tucker K. (2025). Non-homogeneous distribution of inhibitory inputs among motor units in response to nociceptive stimulation at moderate contraction intensity. J. Physiol..

[59] Olson M., Solomonow M., Li L. (2006). Flexion-relaxation response to gravity. J. Biomech..

[60] Vasavada A.N., Peterson B.W., Delp S.L. (2002). Three-dimensional spatial tuning of neck muscle activation in humans. Exp. Brain Res..

[61] McManus L., De Vito G., Lowery M.M. (2020). Analysis and Biophysics of Surface EMG for Physiotherapists and Kinesiologists: Toward a Common Language with Rehabilitation Engineers. Front. Neurol..

